# Re-resection of brain metastases – outcomes of an institutional cohort study and literature review

**DOI:** 10.1186/s12885-025-13677-0

**Published:** 2025-06-01

**Authors:** David Wasilewski, Zoe Shaked, Annalena Fuchs, Siyer Roohani, Ran Xu, Max Schlaak, Nikolaj Frost, Martin Misch, David Capper, David Kaul, Julia Onken, Peter Vajkoczy, Felix Ehret

**Affiliations:** 1 Charité – Universitätsmedizin Berlin, Corporate Member of Freie Universität Berlin and Humboldt-Universität zu Berlin, Department of Neurosurgery, Berlin, Germany; 2 Charité – Universitätsmedizin Berlin, Corporate Member of Freie Universität Berlin and Humboldt-Universität zu Berlin, Charité Comprehensive Cancer Center, Berlin, Germany; 3 Berlin Institute of Health at Charité – Universitätsmedizin Berlin, BIH Biomedical Innovation Academy, BIH Charité (Junior) Clinician Scientist Program, Berlin, Germany; 4 Charité – Universitätsmedizin Berlin, Corporate Member of Freie Universität Berlin and Humboldt-Universität zu Berlin, Department of Radiation Oncology, Berlin, Germany; 5 German Cancer Consortium (DKTK), partner site Berlin, a partnership between DKFZ and Charité – Universitätsmedizin Berlin, Berlin, Germany; 6 Charité – Universitätsmedizin Berlin, Corporate Member of Freie Universität Berlin and Humboldt-Universität zu Berlin, Department of Dermatology, Venereology and Allergology, Berlin, Germany; 7 Charité – Universitätsmedizin Berlin, Corporate Member of Freie Universität Berlin and Humboldt-Universität zu Berlin, Department of Infectious Diseases and Pulmonary Medicine, Berlin, Germany; 8 Charité – Universitätsmedizin Berlin, Corporate Member of Freie Universität Berlin and Humboldt-Universität zu Berlin, Institute of Neuropathology, Berlin, Germany

**Keywords:** Brain metastasis, Resection, Re-resection, Radiotherapy, Breast cancer, Melanoma, Lung cancer, Survival

## Abstract

**Background:**

Surgically accessible brain metastases are treated through microsurgical removal followed by radiation therapy, resulting in improved progression-free and overall survival. Some patients experience recurrence, prompting the need for effective management strategies. Despite the prevalence of recurrence, there remains a gap in the literature regarding the outcomes of patients undergoing re-resection of brain metastases.

**Objectives:**

This study aims to comprehensively characterize clinical, radiological, histopathological, and treatment-related aspects, along with outcomes, for patients undergoing re-resection of locally and distantly recurrent brain metastases.

**Methods:**

We conducted a single-center retrospective cohort study, including patients who underwent secondary brain metastasis resection following prior primary brain metastasis resection and irradiation.

**Results:**

Among 60 patients, 41 (68.3%) had local recurrences, and 19 (31.7%) had distant recurrences. Median intracranial progression-free survival was 7.7 months [95% CI: 6.5–11.2], time to re-resection was 11.6 months [95% CI: 9.1–15.3], and overall survival was 30.8 months [95% CI: 20.4–49.5]. Non-small cell lung cancer (NSCLC) was the most common primary tumor. Post-initial resection treatments included radiation alone (31.7%), radiation plus chemotherapy (25.0%), radiation plus targeted therapy (15.0%), and radiation plus immunotherapy (28.3%). Cavity irradiation was performed in 46 patients (76.7%) and whole brain radiation in 14 (23.3%). Post-re-resection treatments varied: 21 patients (35.0%) received best supportive care, 15 (25.0%) radiation only, 12 (20.0%) systemic therapy only, and 12 (20.0%) both radiation and systemic therapy. Independent risk factors for shorter overall survival included non-breast cancer histology, pre-re-resection tumor volume > 9 mL, pre-re-resection Karnofsky Performance Status ≤ 60%, and presence of vital tumor cells at re-resection.

**Conclusion:**

Brain metastasis resection of local and distant recurrences is feasible and a treatment option for selected patients with good clinical performance status. This study underscores the potential role of re-resection in brain metastasis. Further research to improve patient selection and treatment algorithms is warranted.

**Supplementary Information:**

The online version contains supplementary material available at 10.1186/s12885-025-13677-0.

## Introduction

Brain metastases are a common occurrence in patients with solid malignancies, with up to 50% of such patients developing brain metastases during the course of their disease [1, 2]. Brain metastases can significantly impact the quality of life (QoL) and clinical outcomes of patients. Both QoL and survival can be improved through aggressive treatments, such as microsurgical brain metastasis resection followed by radiotherapy (RT), which are regularly considered for most patients [3–5]. Neurosurgical resection represents one of the main treatment modalities in primary as well as recurrent brain metastases and is indicated in patients with good Karnofsky performance status (KPS), particularly in those with large brain metastases (≥ 30 mm) that are surgically accessible and/or provoke a significant mass effect and associated local edema. Other decision criteria are incipient hydrocephalus or occlusive hydrocephalus as well as significant neurological deficits [1, 4]. Current guidelines recommend additional local therapy with stereotactic radiosurgery (SRS) of the resection cavity or combined SRS and whole brain radiation therapy (WBRT), depending on the extent and localization of remaining intracranial disease [2–6]. The choice of upfront treatment as well as additional treatment modalities depends on a multitude of factors, e.g., KPS, extracranial disease burden, size, localization and number of brain metastases, additional available treatment options, underlying primary cancer, necessity of tissue sampling, etc., and should be discussed in multidisciplinary teams [2–4]. RT consisting of either SRS or WBRT improves intracranial disease control and can prolong intracranial progression-free survival (icPFS) and overall survival (OS) [7–9]. Nonetheless, apart from previously described factors such as the extent of resection (EOR) and operative technique of brain metastasis removal (i.e., piecemeal vs. *en bloc* resection), the ideal management of recurrent brain metastases following initial neurosurgery and consolidative RT is still poorly understood, despite the relatively high prevalence of brain metastasis recurrence, which is quoted in the literature as 20–40% and is likely to increase due to improved local and systemic therapy options and improved follow-up measures [9–11]. One treatment paradigm, repeat resection, might be a valid treatment method in a subset of patients with pre-treated brain metastases that show evidence of disease recurrence on follow-up cranial imaging. Several retrospective studies suggest that a ‘re-do craniotomy’, i.e., re-resection, is a viable treatment option, which is associated with prolonged OS and may also positively impact patients’ QoL, yet a comprehensive characterization of the clinical, radiological, histopathological, and treatment-related aspects, along with outcomes, for patients undergoing re-resection of locally and distantly recurrent brain metastases is lacking [10–15]. In this study, we sought to characterize patients who underwent a microsurgical brain metastasis re-resection following an initial resection and consolidative RT. We provide data on clinical outcomes and review the available literature on brain metastasis re-resection.

## Methods

### Patient selection and study design

**Fig. 1:**
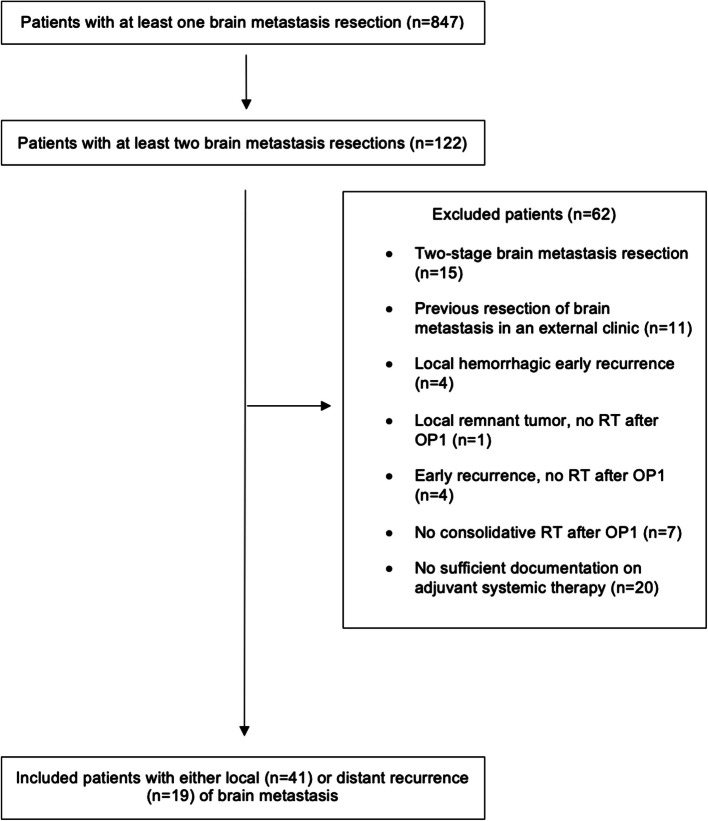
CONSORT diagram of included and excluded patients. Our cohort included 60 patients who underwent a re-craniotomy and recurrent brain metastasis resection for either a local or distant brain metastasis recurrence

Adult patients with diagnosis of a solid, non-nervous-system malignancy and a radiological and/or neuropathological confirmation of brain metastasis treated at one of the three sites of a large tertiary center between January 2010 and February 2023 were identified through our institutional electronic medical records. Exclusion criteria are specified in the CONSORT diagram (Fig. 1). Only patients with a histopathological confirmation of an intracerebral brain metastasis in the context of a primary surgical brain metastasis resection were included. If a patient experienced both a local and a distant recurrence, they were counted as a single case, assigned to the local recurrence group, and analyzed as such. Local recurrences were defined as metastases occurring in direct proximity to the first brain metastasis, whereas distant recurrences were defined as metastases occurring in an anatomical location other than the site of resection (i.e., at least 4 cm from the initial resection cavity).

### Endpoints of interest

The primary endpoints of interest were OS and icPFS. OS was measured from the time of primary brain metastasis resection to the time of death by any cause or the last day of available follow-up. icPFS was defined as the time from the primary brain metastasis resection to the first sign of recurrence on cranial magnetic resonance imaging (cMRI).

### Clinical, radiological and treatment-related characteristics

Baseline characteristics were comparable to previous studies in the field and included clinical, radiological, and histopathological features, as well as treatment-related features [8–11]. The KPS was assessed after both the first brain metastasis resection (OP1) and the re-resection of a recurrent brain metastasis (OP2), dichotomized as good (> 60%) or poor (≤ 60%). Demographic parameters included age, sex, primary cancer entity, type of recurrence, total number of brain metastasis resections, chief complaints before OP1 and OP2, and cause of death (Table 1).

The radiological assessment included anatomical localization of the resected brain metastases, presence of hydrocephalus or hemorrhage, and presence of active extracranial metastases at both OP1 and OP2, based on reports from board-certified radiologists (Table 2). Tumor volumes were quantified using a semi-automated 3D rendering algorithm in iPlannet (Brainlab, Munich, Germany) utilizing the SmartBrush tool. Due to rounding to one decimal place, percentages may not sum up to 100%.

Histopathological evaluation included tumor vitality, the presence of necrotic areas, and the Ki-67 proliferation index of resected brain metastasis tissue from OP1 and OP2 (Table 4). Neuropathological review was mandatory for all patients.

Neurological deficits before and after OP2 included impairments in gait, hand function, coordination, language, and vision (Table 3). Treatment-related features encompassed therapies received before OP1 and those administered postoperatively after both OP1 and OP2. The 2 Gy equivalent dose (EQD2) with an α/β ratio of 10 Gy was calculated (Table 5).

### Statistical analysis

Differences between the groups were assessed using the Wilcoxon rank sum test for continuous and categorical variables, respectively. Matched pair analyses of tumor volumes between OP1 and OP2 and KPS at OP1 and OP2 were done using the Wilcoxon signed-rank test. The Kaplan-Meier estimator was used to analyze OS and icPFS. A reference cohort from our institutional registry, including 539 patients who underwent only a single microsurgical brain metastasis resection in the course of their disease, was used to compare the outcomes of interest with our cohort. Multivariable Cox regression models for OS and icPFS were computed using clinical covariables deemed associated with the respective outcomes. Descriptive statistics, including frequencies, means, and standard deviations (SDs), Kaplan-Meier, and Cox proportional hazards regression analysis as previously described, were computed using R version 4.2.2 (R Foundation) [6]. A p-value < 0.05 was considered significant, with p-values being 2-sided.

### Literature review

A non-systematic literature review on the re-resection of brain metastases was conducted to contextualize our findings within the existing body of evidence. The review focused on studies that analyzed outcomes following repeat resection of recurrent brain metastases, including survival metrics, recurrence patterns, and treatment characteristics.

### Search strategy and study selection

The literature search was conducted using the National Library of Medicine (PubMed) database and Google Scholar, with searches completed on August 1, 2023. The following search terms and Medical Subject Headings (MeSH) terms were used in various combinations:

- “brain metastases”,
- “re-resection”,
- “repeat surgery”,
- “local recurrence”,
- “neurosurgical outcomes”,
- “survival after repeat resection of brain metastases”.

Boolean operators (AND/OR) were applied to refine search results. Filters were set to include English-language articles with full-text availability. No restrictions were placed on publication year to ensure a comprehensive review of the literature.

### Inclusion and exclusion criteria

Studies were included if they:

Specifically analyzed patients undergoing re-resection of recurrent brain metastases.

1. Reported OS, icPFS, or time to recurrence as outcome measures.
2. Provided details on treatment modalities, including systemic therapy and radiotherapy, before and after surgery.

Excluded were:

- Case reports, reviews, or studies focusing solely on single-resection cohorts without re-resection data.
- Studies with insufficient survival or recurrence data.

### Data Extraction and Analysis

Supplementary Tables 1–3 summarize the extracted data, including the number of patients, localization of recurrence, tumor histology, and survival outcomes across studies.

### Ethical approval

The study was approved by the local institutional review board (EA2/121/20). Due to the retrospective design of the study, written informed consent was waived.

## Results

### Patient characteristics

A total of 60 patients with a median age of 59.5 years (46.3–67.1 years; IQR) were included in our cohort, of which 35 were female (58.3%) and 25 were male (41.7%). Patient demographic characteristics are summarized in Table 1. The median time-to-recurrence resection (i.e., the time from the first brain metastasis resection (OP1) to the re-resection (OP2)) was 11.6 months (9.1–15.3; IQR). Median OS (i.e., time from OP1 till death) was 30.8 months [95% CI: 20.4–49.5], and median icPFS (i.e., time from OP1 till first progression) was 7.7 months [95% CI: 6.5–11.2]. Median OS from OP2 till death was 11.0 months [95% CI: 6.4–21.9]. Forty-three patients have died (71.3%) and 17 patients (28.3%) were still alive at the last follow-up. In our cohort, 41 patients (68.3%) have had a local, and 19 patients (31.7%) have had a distant recurrence (Table 1, Fig. 1, Supplementary Fig. 1). Further information on radiological features and histopathological and treatment-related characteristics are shown in Tables 2, 3, 4 and 5. The most common underlying primary cancer was NSCLC with 23 patients (38.3%), followed by breast cancer with 14 patients (23.3%) and melanoma with 13 patients (21.7%) (Table 1). The median time from diagnosis of primary disease to the first brain metastasis resection was 22.2 months (15.3–57.7; IQR).

**Table 1:** Demographic and clinical patient characteristics

Characteristic	*N* = 60
**Age (years)**,** Median (IQR)**	59.5 (46.3–67.1)
**Gender**,** n (%)**	
Female	35 (58.3)
Male	25 (41.7)
**Underlying primary cancer entity**,** n (%)**	
Breast cancer	14 (23.3)
Melanoma	13 (21.7)
NSCLC	23 (38.3)
RCC	3 (5.0)
SCLC	6 (10.0)
Undifferentiated (pulmonary) sarcoma	1 (1.7)
**Chief complaints at baseline (OP1)**,** n (%)**	
Headache	13 (21.7)
Sensory-motor symptoms	9 (15.0)
Seizures	7 (11.7)
Alterations in behaviour	2 (3.3)
Aphasia	6 (10.0)
Vertigo/dyscoordination	12 (20.0)
Headache, nausea, vomiting	2 (3.3)
Visual impairment	3 (5.0)
During staging	2 (3.3)
Incidental or others	4 (6.7)
**KPS after OP1, n (%)**	
≤ 60	5 (8.3)
> 60	55 (91.7)
**KPS after OP2, n (%)**	
≤ 60	9 (15.0)
> 60	51 (85.0)
**Surgical complications (30 days post-OP2)**,** n (%)**	
CSF fistula	1 (1.7)
None	51 (85.0)
Parenchymal bleeding (near the resection cavity)	1 (1.7)
Pneumocephalus	4 (6.7)
Post-operative surgical wound infection	2 (3.4)
Subdural hematoma	1 (1.7)
**Surgical reintervention (30 days post-OP2)**,** n (%)**	
None	58 (96.7)
Wound revision	2 (3.3)
**Medical complications (30 days post-OP2)**,** n (%)**	
ARDS	1 (1.7)
Delirium	2 (3.3)
Diverticulitis	1 (1.7)
None	55 (91.7)
Pneumonia	1 (1.7)
**Cause of death**,** n (%)**	
Alive	17 (28.3)
CNS disease	18 (30.0)
Not known	13 (21.7)
Other (cardiac arrythmias)	1 (1.7)
Other (delirium)	1 (1.7)
Other (pneumonia)	5 (8.3)
Other (septic shock)	1 (1.7)
Systemic disease (primary tumor)	4 (6.7)

General demographic and clinical characteristics at baseline (i.e., at the time of first brain metastasis resection, i.e., OP1) and at re-resection (i.e., OP2), as well as information regarding the cause of death of patients included in our cohort (*n*=60). A good postoperative KPS was defined as > 60%, whereas a KPS ≤ 60% was considered poor. Furthermore, the table provides a detailed overview of postoperative complications within 30 days after the second surgical intervention (OP2)

**Table 2:** Imaging characteristics at baseline (before OP1) and before OP2

Characteristic	*N* = 60
**Type of recurrence**, ***n*** (%)	
Local	41 (68.3)
Distant	19 (31.7)
**Number of brain metastases at baseline**,** n (%)**	
One brain metastasis	34 (56.7)
Two brain metastases	19 (31.7)
Three brain metastases	7 (11.7)
**Number of brain metastases before OP2**,** n (%)**	
One brain metastasis	35 (58.3)
Two brain metastases	14 (23.3)
Three brain metastases	11 (18.3)
**Anatomical site of index brain metastasis at baseline**,** n (%)**	
Frontal	18 (30.0)
Temporal	12 (20.0)
Parietal	10 (16.7)
Occipital	5 (8.3)
Cerebellar	15 (25.0)
Other	0 (0.0)
**Anatomical site of index brain metastasis before OP2**,** n (%)**	
Frontal	14 (23.3)
Temporal	11 (18.3)
Parietal	11 (18.3)
Occipital	6 (10.0)
Cerebellar	17 (28.3)
Other	1 (1.7)
**Hydrocephalus at baseline**,** n (%)**	
No hydrocephalus	53 (88.3)
Hydrocephalus	7 (11.7)
**Hemorrhage at baseline**,** n (%)**	
No hemorrhage	42 (70.0)
Hemorrhage	18 (30.0)
**Leptomeningeal disease at baseline**,** n (%)**	
No LMD	58 (96.7)
LMD	2 (3.3)
**Localization of (all) intracranial metastases at baseline**,** n (%)**	
Supratentorial	44 (73.3)
Infratentorial	9 (15.0)
Both	7 (11.7)
**Localization of (all) intracranial metastases before OP2**,** n (%)**	
Supratentorial	41 (68.3)
Infratentorial	13 (21.7)
Both	6 (10.0)
**Extracranial metastases before OP1**,** n (%)**	
No extracranial metastases	41 (68.3)
Presence of extracranial metastases	18 (30.0)
Unknown	1 (1.7)
**Extracranial metastases before OP2**,** n (%)**	
No active extracranial metastases	41 (68.3)
Presence of active extracranial metastases	11 (18.3)
Unknown	8 (13.3)
**Tumor volume at baseline**,** Median (IQR)**	17.3 (9.1–30.1)
Unknown	10
**Tumor volume before OP2**,** Median (IQR)**	7.3 (4.1–13.7)
Unknown	3

Imaging characteristics of our cohort at baseline (i.e., OP1) and at re-resection (i.e., OP2) (*n*=60). CT staging imaging was missing for one patient at the time of the first brain metastasis resection (OP1) and missing for eight patients in the context of the second brain metastasis resection (OP2). In 10 patients, there was no pre-operative volume measurement available from before the first brain metastasis resection (OP1). In 3 cases, there was no information on the tumor volume prior to OP2

**Table 3:** Neurological deficits before and after re-resection (OP2)

Variables (*n* = 60)	Before re-resection (OP2)	After re-resection (OP2)
**Neurological deficit**, ***n*** (%)		
Yes	43 (71.7)	40 (66.7)
No	17 (28.3)	19 (31.7)
Unknown	0 (0.0)	1 (1.7)
**Type of neurological deficit**,** n (%)**		
**Gait**		
Yes	27 (45.0)	20 (33.3)
No	33 (55.0)	39 (65.0)
Unknown	0 (0.0)	1 (1.7)
**Hand function**		
Yes	21 (35.0)	20 (33.3)
No	39 (65.0)	39 (65.0)
Unknown	0 (0.0)	1 (1.7)
**Language**		
Yes	7 (11.7)	9 (15.0)
No	53 (88.3)	50 (83.3)
Unknown	0 (0.0)	1 (1.7)
**Behavior**		
Yes	3 (5.0)	2 (3.3)
No	57 (95.0)	57 (95.0)
Unknown	0 (0.0)	1 (1.7)
**Orientation**		
Yes	2 (3.3)	1 (1.7)
No	57 (95.0)	59 (98.3)
Unknown	1 (1.7)	0 (0.0)
**Vision**		
Yes	10 (16.7)	12 (20.0)
No	49 (81.7)	47 (78.3)
Unknown	1 (1.7)	1 (1.7)
**Coordination**		
Yes	10 (17.5)	6 (10.0)
No	49 (81.7)	53 (88.3)
Unknown	1 (1.7)	1 (1.7)

Information regarding the presence of neurological deficits before and following brain metastasis re-resection in our cohort (*n*=60). Neurological deficits were classified as problems with gait, hand function, language, behavior, orientation, vision, and coordination. Following OP2, four patients who previously had a neurological deficit became deficit-free, and two patients who did not have a deficit preoperatively developed a deficit after surgery. The orientation, vision, and coordination of 1 patient could not have been examined pre-operatively due to an impairment in consciousness. The presence of neurological deficits could not be assessed in 1 patient postoperatively due to their death shortly after the operation

**Table 4:** Histopathological patient characteristics

Characteristic	*N* = 60
**Vital tumor cells from resected tissue (OP1)**, ***n*** (%)	
No vital tumor tissue	3 (5.0)
Vital tumor tissue	57 (95.0)
**Vital tumor cells from resected tissue (OP2)**,** n (%)**	
No vital tumor tissue	11 (18.3)
Vital tumor tissue	49 (81.7)
**Ki67 index (%) (from OP1)**,** Median (IQR)**	30.0 (20.0–50.0)
Unknown	7
**Ki67 index (%) (from OP2)**,** Median (IQR)**	30.0 (27.5–50.0)
Unknown	15
**Necrosis in tissue described by neuropathologist (OP1)**,** n (%)**	
No necrosis	5 (8.3)
Necrosis	30 (50.0)
Unknown or not explicitly described	25 (41.7)
**Necrosis in tissue described by neuropathologist (OP2)**,** n (%)**	
No necrosis	2 (3.3)
Necrosis	47 (78.3)
Unknown or not explicitly described	11 (18.3)

Histopathological characteristics of the study’s cohort (*n*=60). Information about the vitality of resected tumor tissue and reporting of Ki67 from each operation primary resection (OP1) and secondary resection (OP2) are listed. The Ki67 index was not reported in seven cases upon primary brain metastasis resection and in 15 cases in the context of the secondary resection. This was, in part, a consequence of the presence of radionecrosis in the tissue specimens resected during brain metastases re-resection (OP2)

**Table 5:** Treatment-related patient characteristics before primary brain metastasis resection (OP1), after OP1) and after re-resection (OP2)

Characteristic	*N* = 60
**Total number of brain metastasis resections**, ***n*** (%)	
Two	53 (88.3)
Three	5 (8.3)
Four	1 (1.7)
Five	1 (1.7)
**Primary tumor resection**,** n (%)**	
No primary tumor resection	18 (30.0)
Primary tumor resection before OP1	38 (63.3)
Primary tumor resection after OP1	4 (6.7)
**Systemic therapies before OP1**,** n (%)**	
Naive at OP1	20 (33.3)
Systemic pre-treatment before OP1	40 (66.7)
**Cranial irradiation before OP1**,** n (%)**	
Naive for cranial irradiation at OP1	48 (80.0)
Pre-treatment with cranial irradiation before OP1	12 (20.0)
**Systemic pre-treatment OP1**,** n (%)**	
No therapy	20 (33.3)
CPIs or combined CPI and RT	10 (16.7)
CTx or combined CTx and RT	25 (41.7)
TT or combined TT and RT	5 (8.3)
**Adjuvant systemic treatment after OP1**,** n (%)**	
RT only	19 (31.7)
RT + CTx	15 (25.0)
RT + TT	9 (15.0)
RT + CPIs	17 (28.3)
**Type of radiation therapy after OP1**,** n (%)**	
FSRT	23 (38.3)
Intraoperative RT	1 (1.7)
Conventional RT	11 (18.3)
SRS	11 (18.3)
WBRT	14 (23.3)
**Target of consolidative RT after OP1**,** n (%)**	
Resection cavity	46 (76.7)
WBRT	14 (23.3)
**Total dose after OP1 (Gy)**,** Median (IQR)**	30.0 (24.0–33.0)
**Dose per fraction after OP1 (Gy)**,** Median (IQR)**	3.0 (3.0–8.0)
**Fractions after OP1**,** Median (IQR)**	10.5 (3-11)
**EQD2 (alpha/beta = 10)**,** Median (IQR)**	36.0 (33.1–42.3)
**Residual tumor after OP1**,** n (%)**	
No residual tumor	26 (43.3)
Residual tumor	12 (20.0)
Unknown	22 (36.7)
**Adjuvant systemic treatment after OP2**,** n (%)**	
Best supportive care	21 (35.0)
Local +/- systemic treatment after OP2	39 (65.0)
**Adjuvant systemic treatment after OP2**,** n (%)**	
Best supportive care	21 (35.0)
CPI	7 (11.7)
CTx	1 (1.6)
RT + CPI	7 (11.7)
RT + CTx	2 (3.3)
RT + TT	3 (5.0)
RT only	15 (25.0)
TT	4 (6.7)
**Type of radiation therapy after OP2**,** n (%)**	
FSRT	8 (13.3)
No radiation therapy	33 (55.0)
Conventional RT	2 (3.3)
SRS	8 (13.3)
WBRT	9 (15.0)
**Target of consolidative RT after OP2**,** n (%)**	
Irradiation of resection cavity or a brain metastasis	18 (30.0)
WBRT	9 (15.0)
**Total dose after OP2 (Gy)**,** Median (IQR)**	24.0 (21.0–30.8)
**Dose per fraction after OP2 (Gy)**,** Median (IQR)**	4.0 (3.0–15.0)
**Fractions after OP2**,** Median (IQR)**	5 (1-11)
**EQD2 (alpha/beta = 10) after OP2**,** Median (IQR)**	35.8 (31.3–42.0)
**Residual tumor after OP2**,** n (%)**	
No residual tumor	29 (48.3)
Residual tumor	8 (13.3)
Unknown	23 (38.3)

Treatment-related characteristics of the cohort (*n*=60). Forty-eight patients (80%) did not undergo RT before the initial resection, whereas 20 patients (33.3%) did not receive any systemic treatment prior to the primary resection. Of the 42 patients (66.7%) who did receive systemic treatment, the majority received chemotherapy (CTx), followed by checkpoint inhibition (CPI) and targeted therapy (TT). Treatment-related characteristics of the cohort (*n*=60). Therapy after OP1 included either RT only, a combination of RT and CTx, a combination of RT and TT, or a combination of RT and CPIs. Intraoperative radiation was delivered to one patient. Patients who received RT and CPIs (*n*=17) received either Nivolumab (*n*=6), Nivolumab/Ipilimumab combinations (*n*=3), Pembrolizumab (*n*=3), Atezolizumab (*n*=1) or other combinations of different checkpoint inhibitors and immunotherapy (*n*=4). Therapy after OP2 included a variety of different modalities and combinations thereof. The most common treatment regimens following re-resection included RT only (25.4%), CPI only (11.1%), and a combination of RT and CPI (7.9%). Ten patients did not receive any further local or systemic therapy after OP2. Eight patients received multiple separate sessions of radiation therapy following re-resection. The total dose and the number of fractions for these patients were calculated by summing the individual total doses or number of fractions, respectively. The dose in Gy per fraction was calculated by dividing the sum of the total doses by the sum of the number of fractions of all sessions. Six patients received multiple types of radiation therapy following OP2; of those, 2 patients received a combination of FSRT and conventional RT, 1 patient received a combination of SRS and conventional RT, and 3 patients received a combination of SRS and FSRT

At the time of the initial brain metastasis diagnosis, 34 patients (56.7%) had one brain metastasis, 19 patients (31.7%) had two brain metastases, and 7 patients (11.7%) had more than two brain metastases. The dominant or index brain metastases were most frequently located in the frontal (30.0%), cerebellum (25.0%), temporal region (20.0%) (Table 2). Similarly to baseline, most patients (58.3%) showed a single brain metastasis at recurrence. Yet, the number of patients with two brain metastases decreased (from 31.7% before OP1 to 23.3% at re-resection) whilst the number of patients with > 2 metastases increased (from 11.7 to 18.3%) (Table 2). The most common site of the index metastasis at OP2 was the cerebellum with 28.3%.

The most common leading symptoms before OP1 were headache (13 patients, 21.7%), cerebellar symptoms (12 patients, 20.0%), and sensory-motor symptoms (9 patients, 15.0%) (Table 1). Index brain metastasis of the patients in our cohort showed that the metastases were significantly larger at baseline, i.e., before OP1, compared to the recurrent lesion at OP2, both in patients with local and distant recurrences (*p* < 0.001) (Table 2).

Specific symptoms before and after re-resection (OP2) are shown in Table 3. Following the re-resection, four patients who previously had a neurological deficit became deficit-free, and two patients who did not have a deficit previously acquired one. In total, 66.7% of patients had some type of neurological deficit after re-resection (OP2). The most common neurological deficits following re-resection were deficits in gait and the hand function (33.3% of patients, respectively), followed by vision (20.0%) and language deficits (15.0%) (Table 3). Despite the relatively high percentage of patients with neurological deficits, 85.0% of patients had a good clinical status (i.e., KPS > 60%) following re-resection. Following re-resection, 9 patients (15.0%) suffered from some surgical complications, such as pneumocephalus (4 patients), bleeding (2 patients), surgical wound infections (2 patients), or cerebrospinal fluid fistula (1 patient), whereas 5 patients (8.3%) developed a medical complication, such as delirium or acute respiratory distress (Table 1).

Vital tumor cells were detected in 95.0% of tissue specimens resected during OP1 and showed a Ki67 index of 30% on average (20.0–50.0; IQR). In contrast to the first resection, vital tumor cells were detected in only 81.7% of the resected tissue specimens with a median Ki67 index of metastases resected during re-resection was, at 30.0% (27.5–50.0; IQR) (Table 4). Importantly, the rate of neuropathologically described necrosis was considerably higher in the resected tissue of OP2 (78.3% vs. 50.0%).

Before the initial resection (OP1), 12 patients (20.0%) received cranial irradiation, and in total, 40 patients (66.7%) received systemic therapy before OP1, whereas twenty patients (33.3%) were entirely treatment-naïve (Table 5).

**Fig. 2:**
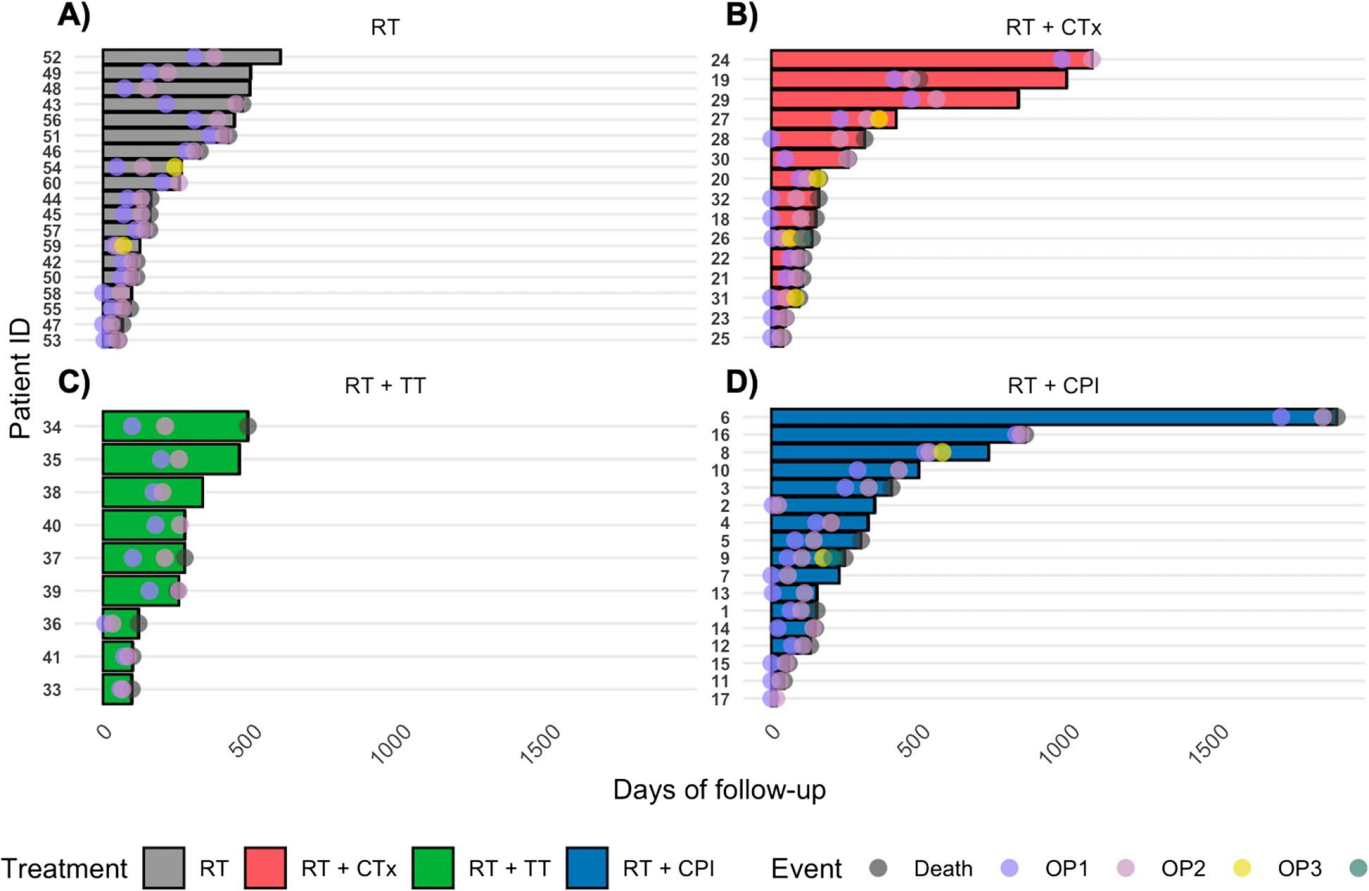
Swimmer plots of distinct adjuvant treatment subgroups. Swimmer plots showing the follow-up of patients grouped according to the type of adjuvant systemic treatment after the first brain metastasis resection (OP1): **A**) RT only, **B**) RT and CTx, **C**) RT and TT (with antibodies in case of breast cancer, i.e., mainly Trastuzumab with or without Emtansin or small molecule inhibitors, i.e., kinase inhibitors mainly in melanoma) and **D**) RT and CPI. All patients received at least 2 cycles of systemic therapy after OP1 or before OP2, respectively

Nineteen patients (31.7%) received RT only, 15 patients (25%) received RT and chemotherapy (CTx), 9 patients (15.0%) were treated with RT and targeted therapy (TT), and 17 patients (28.3%) received RT in combination with checkpoint inhibition (CPI) (Table 5, Fig. 2). As for adjuvant RT after OP1, most patients received fractionated stereotactic radiotherapy (FSRT) with 23 patients (38.3%), followed by WBRT in 14 patients (23.3%) and conventional RT with 11 patients (18.3%) and SRS with 11 patients (18.3%), respectively. One patient received intraoperative RT. Following OP1, 46 patients (76.7%) received RT of the resection cavity, whereas 14 patients underwent WBRT (23.3%) (Table 5). In at least 26 cases (43.3%), a gross total resection (GTR) was achieved at OP1, whereas at the time of re-resection, GTR was achieved in at least 29 cases (48.3%) (Table 5).

In terms of local or systemic treatment following re-resection (OP2), 21 patients (35.0%) received no further local or systemic treatment, i.e., best supportive care (BSC), whereas 12 patients (20.0%) received RT and systemic therapies after OP2 and 15 patients (25.0%) only RT without systemic treatment. The most common RT modalities were WBRT (15.0%), followed by SRS (13.3%) and FSRT (13.3%) (Table 5). Patients who received no systemic treatment after OP2 did so for various reasons, such as good control of primary disease (i.e., remission), patient preferences, and medical comorbidities (data not shown).

### Prognostic factors

**Fig. 3:**
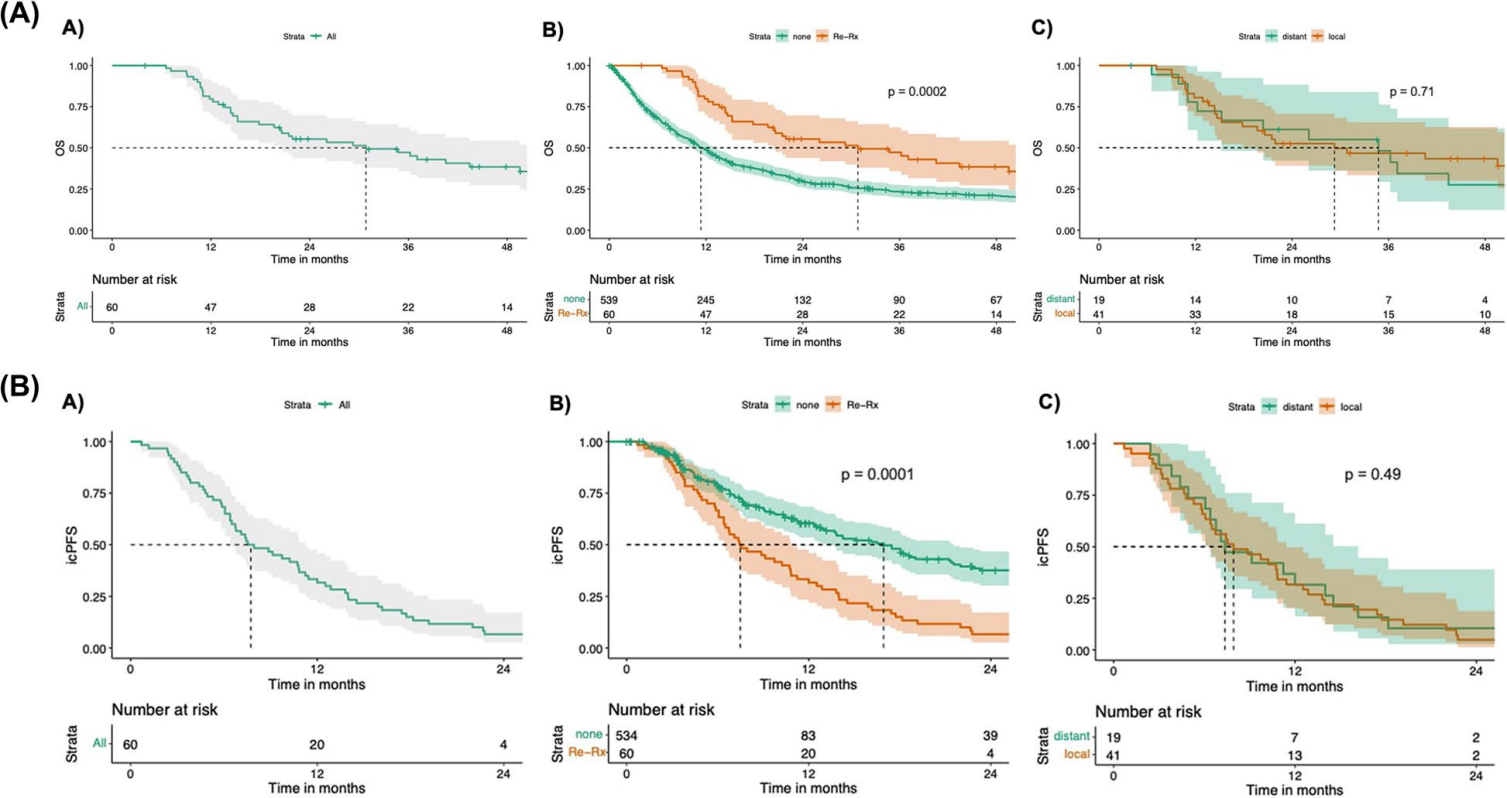
Kaplan-Meier estimates for survival analysis. Kaplan-Meier curves and associated risk table displaying **(A)**, A) the median OS of the total cohort of interest (*n*=60), **(A)**, B) a comparison of both subgroups of patients with a group of patients from our registry (reference cohort; *n* =539), in which all patients underwent a single brain metastasis resection; **(A)**, C) the cohort of interest divided into two subgroups; patients with either local or distant brain metastasis recurrence. Kaplan-Meier estimates for analysis of icPFS. Kaplan-Meier curves and associated risk table displaying median time to intracranial progression (icPFS) **(B)**, A) of the total cohort of interest (*n*=60), **(B)**, B) patients with either local or distant brain metastasis recurrence. Both subgroups of patients were compared with respect to icPFS with a group of patients from our registry, in which all patients underwent a single brain metastasis resection, serving as the control group; **(B)**, C) the cohort divided into two subgroups

**Fig. 4:**
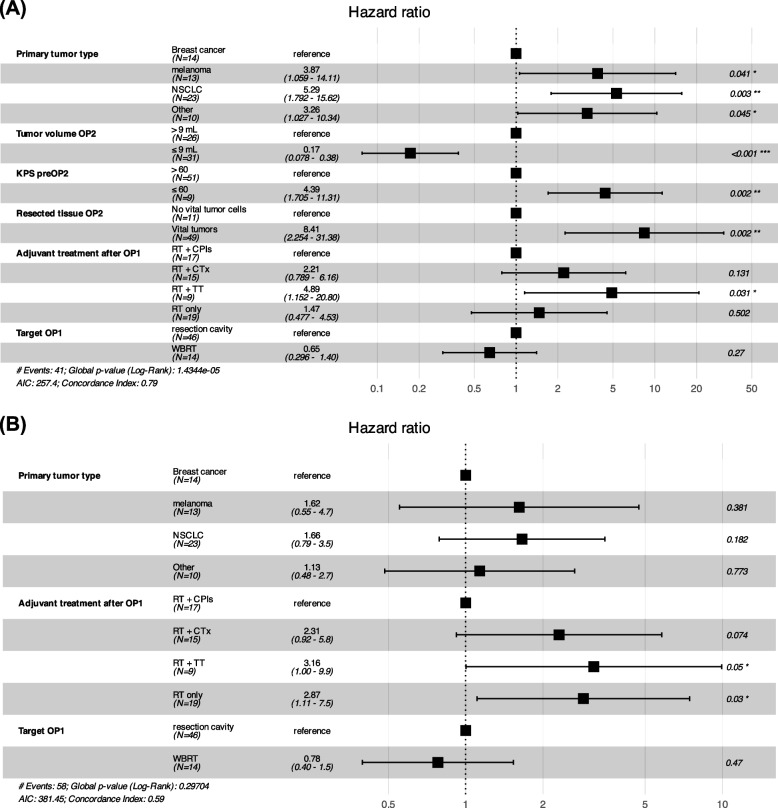
Forest plot of overall survival (OS) using Cox proportional hazard model **A**). Forest plot (hazards plot) reporting the hazard ratio (HR) and 95% confidence intervals of the HR for OS in patients with brain metastasis recurrence; covariables tested/used in the Cox model are visualized on the left side with bolded letters, whereas the vertical dashed line signifies a hazard ratio of 1.0. The discrepancy between the total number of events (43 deaths) and the 41 events included in the Cox model arises because patients with missing data in any of the model's variables were excluded from the analysis (complete case analysis). Forest plot of intracranial progression-free survival (icPFS) **B**) using Cox proportional hazard model. Forest plot reporting the hazard ratio (HR) and 95% confidence intervals of the HR for the icPFS in patients with brain metastasis recurrence; covariables tested in the Cox model are depicted on the left side with bolded letters, whereas the vertical dashed line signifies a hazard ratio of 1.0. Only mechanistically relevant factors including systemic treatment and type of radiation therapy aside from primary tumor type were included in this model

The median follow-up of the total cohort was 53.0 months (38.2–79.6; IQR). The time from OP1 to death, i.e., the median OS for the total cohort was 30.8 months [95% confidence interval (CI): 20.4–49.5], and the median time from OP2 to death was 11.0 months [95% CI: 5.6–20.4]. The OS of patients undergoing resection of local brain metastasis recurrence was 29.3 months [95% CI: 17.8 – not estimable (NE)], whereas the OS of patients undergoing resection of distant brain metastasis recurrence was 34.7 months [95% CI: 15.2 – NE] (Fig. 3). Despite undergoing multiple brain metastases resections, 18 patients (30.0%) died due to CNS disease. In comparison, only 3 patients (5.0%) died from their systemic disease, and 12.7% died due to other causes such as pneumonia and multiple organ dysfunction syndrome (Table 1). A reference cohort of 539 patients from our institutional registry, in which each patient underwent only one operative brain metastasis resection in the course of their disease, had a median OS of 11.4 months [95% CI: 10.2–13.2] during a similar observation period to that of the cohort of patients undergoing brain metastasis re-resections we report on here (*p* = 0.0002) (Fig. 3(B), A). NSCLC histology (*p* = 0.003), melanoma histology (*p* = 0.041) or other tumor entities (*p* = 0.043), tumor volume ≤ 9 mL prior to OP2 (*p* < 0.001), KPS ≤ 60% before OP2 (*p* = 0.002), as well as the presence of vital tumor tissue at OP2 (*p* = 0.002) were independently associated with OS. Additionally, adjuvant treatment after OP1 with RT + TT was significantly linked to OS (*p* = 0.031) (Fig. 4a). As for icPFS, adjuvant treatment after OP1 with RT only (*p* = 0.030) and RT + TT (*p* = 0.050) were negatively associated with icPFS as compared to patients receiving RT + CPI after OP1 (Fig. 4b).

## Discussion

Recurrent brain metastases pose a complex clinical problem with increasing incidence and frequently necessitate an interdisciplinary discussion and individualized approach with respect to diagnostic work-up and treatment [1, 8, 10, 12, 15]. There are currently no evidence-based guidelines which can be used to aid clinical decision-making regarding recurrent brain metastasis resection, as all published studies on this matter are retrospective, based on single-center reports, and do not provide comprehensive information on the treatment modalities applied between the primary and secondary brain metastasis resections and detailed follow-up after re-resection [3, 4].

In a non-systematic literature review on the re-resection of brain metastases we have conducted, we have identified a total of five single-center, retrospective studies studying this topic. The first study reporting on re-resection of brain metastases was published in 1995 by Bindal et al., before the widespread use of SRS and modern pharmacologic treatment strategies [10]. The study included 48 patients with recurrent brain metastases (Supplementary Tables 1–3), of which a total of 37 patients also received WBRT; 31 patients after the initial resection and 6 following re-resection. Importantly, Bindal et al. provided data on both icPFS and survival time after re-resection. The icPFS reported in this study was similar to that in a study by Kennion and Hollimann and our study [13]. Kennion and Holliman reported data on 29 patients with recurrent brain metastases [13]. As in our study, they distinguished between icPFS and time-to-recurrence resection. The time-to-resection in Kennion and Holliman’s cohort was 8.3 months (0.8–46.2, IQR), which is shorter than the time-to-recurrence resection of 11.6 months in our study (Supplementary Table 2). In 2013, Schackert et al. reported their experience on re-resection of brain metastases analyzing 67 patients [12]. In the study, detailed information regarding the anatomical locations of the recurrences was reported, yet information on systemic therapy after the first brain metastasis resection was not provided. Notably, in contrast to the other studies, Schackert et al. did report on the treatments completed after re-resection. Recently, Heßler et al. reported their results on 107 patients with recurrent brain metastases, of which 44 underwent re-resection [15]. While detailed information on the RT is provided, the authors did not elaborate on the administered systemic treatments and did not provide information on whether recurrence was local or distant. Their reported median OS of 11.1 months is comparable to the OS data from Bindal et al. (11.5 months) and our cohort. Lastly, Tewarie et al. reported results on 161 patients with local recurrences, characterizing a wide spectrum of primary tumor types and systemic therapies before re-resection [14]. This study’s distinction lies in its inclusion of patients who have received CPI and TT before re-resection, although no data on the OS of these patients were reported (Supplementary Table 2).

In this study, we provide a comprehensive characterization of 60 patients who underwent primary brain metastasis resection at a tertiary center, all receiving consolidative RT with or without additional systemic treatment, and subsequently developed a brain metastasis recurrence, which was addressed with a re-do craniotomy and resection at our institution. Particularly noteworthy is the fact that almost one-third of patients did not receive any systemic therapy between the first brain metastasis resection (OP1) and re-resection (OP2), in most cases due to the absence of extracranial disease activity and patient preferences. The decision to perform a re-resection for brain metastasis recurrence at our institution included consideration of tumor size and location, symptom burden as well as performance status (KPS). Most patients underwent one re-resection. Multiple re-resections were considered for patients in good clinical condition, with stable extracranial disease, recurrent hemorrhagic metastases, and based on patient preferences. Our strict exclusion criteria render this cohort a heavily selected but unique cohort of brain metastasis patients with well-defined adjuvant treatments. Similarly to other studies on brain metastases, NSCLC represented the most common underlying primary cancer [13, 14]. Interestingly, the median OS of our cohort was much longer than the median OS rates of previously reported cohorts of pre-treated recurrent brain metastases patients, which reported a median OS of approximately 7.4–11.5 months [10–15]. One potential explanation for this deviation could be the recent advancements in systemic therapy modalities, which were unavailable when older studies were conducted. Nonetheless, a recently published study by Heßler et al., which evaluated the survival of brain metastasis recurrence patients treated with TT and SRS amongst other modalities, reported a median OS of 11.1 months too [15].

In our study, a reference cohort of 539 patients from our registry who underwent a single operative brain metastasis resection was compared with this study’s cohort. While the patients in the re-resection cohort had a shorter icPFS, they had an insignificantly longer extracranial PFS (ecPFS) and a significantly longer OS (data not shown). This indicates that, at least in our cohort, patients who underwent a brain metastasis re-resection survived significantly longer given a stable extracranial disease situation, as intracranial disease relapse could be tackled via re-craniotomy and re-resection. Another factor which could have potentially contributed to the longer OS of these patients is their probably better clinical status, which played a role in their qualification for another resection. Moreover, it is also plausible that the OS was longer in the re-resection cohort in comparison to the single resection cohort due to immortal time bias, as patients in the re-resection cohort must have survived long enough to undergo a re-resection, inherently introducing a period during which they couldn’t have experienced events such as death.

In this study, we also report icPFS, a metric that is often underreported in clinical studies utilizing real-world data but offers valuable insights into the optimal postoperative management of this patient cohort and the timing of repeat resections (OP2). The observed difference between icPFS (7.7 months) and the interval from OP1 to OP2 (11.6 months) was 3.9 months, suggesting that, upon detecting a potential recurrence, treating physicians frequently opted to await an additional follow-up cMRI (approximately 3 months later) to confirm the diagnosis of recurrent intracranial disease and guide the decision for re-resection.

In a small part of these cases, positron emission tomography (PET) cMRI imaging was done to aid the differentiation between radionecrosis and brain metastasis recurrence (data not shown). As demonstrated by the relatively high proportion of asymptomatic patients upon recurrence diagnosis, standardized and consequent follow-up imaging seems to play an important role in identifying recurrence whilst patients still have a good clinical status, maximizing their chances of being considered for a re-resection and receiving adequate non-surgical treatment promptly. Since we included patients treated within a fairly long period (between January 2010 and February 2023), in which the standards of practice, for example, in terms of imaging and treatment modalities used, may have changed, there is variability in the follow-up characteristics within our cohort. Additionally, there was no available early postoperative cMRI for many of our patients in our cohort for both OP1 and OP2, given that standard post-operative cMRI after resection of a brain metastasis was established in 2021 in our institution. As a consequence, our study fails to provide insights into the value of GTR in this patient cohort. Furthermore, although we started collecting data prospectively as of March 2021, most patients in this cohort were not monitored by cMRI and computed tomography (CT) staging every 3 to 6 months. Therefore, we were unable to provide complete data on ecPFS – an important factor that is known to impact OS in brain metastasis patients [16]. As for intracranial control or time-to-recurrence of brain metastasis, only two studies reported on factors influencing disease recurrence: data from Schackert et al. show that WBRT may prolong time-to-recurrence, whereas our study demonstrated that CPI may have a protective role in terms of intracranial progression [12]. Apart from survival outcomes, in contrast to previous studies, we provide relatively detailed information regarding administered systemic therapy and RT prior to and following the first brain metastasis resection (OP1) as well as after re-resection (OP2).

On multivariable Cox regression analysis, the presence of non-breast cancer histology, pre-re-resection tumor volume > 9 mL before OP2, KPS ≤ 60% before OP2, and the presence of vital tumor cells at re-resection were associated with an increased hazard for death. Adjuvant RT + CPI after OP1 seems to be associated with a decreased hazard for death in comparison to other treatment modalities after OP1 in our cohort, which is supported by data on icPFS. The type of RT after OP1 was, however, not an independent factor for outcome.

Previous studies we gathered in the literature review reported different predictors of OS and icPFS or time-to-recurrence, including the presence of extracranial disease, KPS, age, primary cancer entity, as well as localization and EOR. This might be in part due to the selected prognostic model of each study. Similarly to the findings of our study, which show that the presence of non-breast cancer histology was associated with shorter OS, the primary cancer entity seems to play an important role in OS across the studies included in the literature review [10–15], whereas other covariables seem to be of less importance.

The main limitations of our study include its retrospective nature, small sample size, and the lack of standardized follow-up due to strict inclusion and exclusion criteria aimed at maintaining a homogenous cohort. Despite these limitations, our findings can contribute valuable insights into the treatment of recurrent brain metastasis, highlighting the potential benefits of re-resection for selected patients with good performance status and limited extracranial disease burden. This study does not establish general treatment recommendations but underscores the need for further research involving a larger, more diverse patient population across different clinical settings. Future studies should explore the development of intra- and extracranial disease in more detail, assess the effectiveness of RT and various systemic treatments, and utilize prospective registry data to develop refined treatment algorithms [16–19].

## Conclusion

Brain metastasis re-resection in the context of recurrent brain metastases represents a viable treatment option and is likely to become increasingly frequent in a selected group of patients. Herein, we provide descriptive data on secondary brain metastasis resections in a cohort of well-characterized patients. Future studies are warranted to verify the observed findings and determine the efficacy and safety of this approach in larger cohorts.

## Supplementary Information

Additional file 1. Supplementary Figure 1. Graphical abstract of the study. The patient cohort investigated is summarized; Patients underwent a first brain metastasis resection (OP1) and subsequently received adjuvant treatment with radiotherapy with or without adjuvant systemic therapy (see Figure 2). Patients included in this study presented with either distant or local disease recurrence and underwent another brain metastasis resection (OP2), i.e., resection of either local or distant brain metastasis recurrence

Additional file 2. Supplementary Figure 2. Representative cranial MR imaging of recurrent brain metastases. Representative pre- and postoperative cranial MR imaging of a patient that underwent a resection of a cerebellar brain metastasis secondary to NSCLC **A)** with postoperative cMRI scan **B)** and experienced a distant disease relapse approximately 4 months after the initial surgery in the right temporal region with marked perifocal edema **C).** The patient underwent a re-resection **D)**. In another patient, MR imaging shows an occipital NSCLC brain metastasis **E)**. The postoperative cMRI following the resection shows gross total resection **F)**. The patient experienced a local relapse of his NSCLC brain metastasis approximately 6 months later **G)** and underwent successful re-resection** H**).

Additional file 3. Supplementary Table 1. Literature review on re-resection of recurrent brain metastases. Literature review of published studies (*n*=6 including this study) in the field of recurrent brain metastases that were treated with secondary resection, including details of various treatment characteristics. All studies were single-center studies.

Additional file 4. Supplementary Table 2. Literature review of studies on secondary resection of recurrent brain metastases. Literature review of published studies (*n*=6 including this study) in the field of recurrent brain metastases that were treated with secondary resection, including details of various treatment characteristics. All studies were single-center studies.

Additional file 5. Supplementary Table 3. Literature review of studies on secondary resection of recurrent brain metastases. Literature review of published studies (*n*=6 including this study) in the field of recurrent brain metastases that were treated with secondary resection, including details of various treatment characteristics. All studies were single-center studies.

## Data Availability

The datasets used and/or analyzed during the current study are available from the corresponding author upon reasonable request.
