# Supplementary material for: Re-resection of brain metastases – outcomes of an institutional cohort study and literature review
Source: BMC Cancer. 2025 Jun 1;25:973. doi: 10.1186/s12885-025-13677-0 (PMC12128291; doi:10.1186/s12885-025-13677-0)
Supplement: Supplementary file 3 — Additional file 3. Supplementary Table 1. Literature review on re-resection of recurrent brain metastases. Literature review of published studies (n=6 including this study) in the field of recurrent brain metastases that were treated with secondary resection, including details of various treatment characteristics. All studies were single-center studies. [file 12885_2025_13677_MOESM3_ESM.docx]

| **Publication** | **Number of patients** | **Number of patients that underwent re-resection** | **Localization of recurrence (number of patients)** |  |  | **Entity of primary tumors (in absolute numbers)** |
| --- | --- | --- | --- | --- | --- | --- |
| Bindal et al., Journal of Neurosurgery, 1995 | 48 patients | 48 patients | Local recurrence (30), distant recurrence (16), local and distant recurrence (2) |  |  | Melanoma (14), lung (11), breast (10), kidney (4), colorectal (4), sarcoma (1), germ cell (1), CUP (3) |
| Schackert et al., Acta Neurochirurgica, 2013 | 67 patients | 67 patients | Local recurrence (35), distant recurrences (13), local and distant recurrences (19) |  |  | Melanoma (12), breast (11), colorectal (11), NSCLC (10), CUP (9), kidney (5), bladder (4), cervix (2), ovarian (1), stomach (1), testes (1) |
| Kennion and Holliman, British Journal of Neurosurgery, 2017 | 29 patients | 29 patients | Local recurrences (26), distant recurrences (3) |  |  | Lung (10), breast (7), colorectal (5), esophageal (2), kidney (1), ovarian (1), skin (1), testes (1), thyroid (1) |
|  |  |  |  |  |  |  |
| Heßler et al., BMC Cancer, 2022 | 107 patients | 44 patients | Not specified |  |  | NSCLC (40), breast (21), melanoma (14), gastro-intestinal (11), other (21) |
|  |  |  |  |  |  |  |
|  |  |  |  |  |  |  |
| Tewarie et al., World Neurosurgery, 2022 | 161 patients (170 brain metastases) | Unclear | Local recurrences (161 patients i.e. 170 metastases) |  |  | Lung (65), breast (39), melanoma (33), other (33) |
|  |  |  |  |  |  |  |
| Wasilewski et al., 2023 | 60 patients | 60 patients | Local recurrences (41), distant recurrences (19) |  |  | NSCLC (40), breast (14), melanoma (13), SCLC (6), renal cell carcinoma (3) |
