# Supplementary material for: Re-resection of brain metastases – outcomes of an institutional cohort study and literature review
Source: BMC Cancer. 2025 Jun 1;25:973. doi: 10.1186/s12885-025-13677-0 (PMC12128291; doi:10.1186/s12885-025-13677-0)
Supplement: Supplementary file 4 — Additional file 4. Supplementary Table 2. Literature review of studies on secondary resection of recurrent brain metastases. Literature review of published studies (n=6 including this study) in the field of recurrent brain metastases that were treated with secondary resection, including details of various treatment characteristics. All studies were single-center studies. [file 12885_2025_13677_MOESM4_ESM.docx]

| **Publication** | **Radiotherapy before initial resection (number of patients)** | | **Systemic therapy between initial and re-resection (number of patients)** |  |  | **Radiotherapy between initial and re-resection (number of patients)** | **Systemic therapy after re-resection (number of patients)** | **Radiotherapy after re-resection (number of patients)** |
| --- | --- | --- | --- | --- | --- | --- | --- | --- |
| Bindal et al., Journal of Neurosurgery, 1995 | Not specified | | Not specified |  |  | WBRT (31) | Not specified | WBRT (6) |
| Schackert et al., Acta Neurochirurgica, 2013 | Not specified | | CTx (9) |  |  | WBRT (25) | CTx (5), CTx + WBRT (9) | WBRT +/- further treatment modalities (31) |
| Kennion and Holliman, British Journal of Neurosurgery, 2017 | WBRT (1) | | CTx (6), CTx and WBRT (4), CTx, WBRT and SRS (1) |  |  | CTx and SRS (1), WBRT (8), CTx and WBRT (4), WBRT | CTx or CTx and WBRT (8) | Not specified |
|  |  |  |  |  |  |  |  |  |
| Heßler et al., BMC Cancer, 2022 | One or multiple of WBRT (30), focal RT (24), SRS (53) and brachytherapy (8) | | Not further specified systemic therapy (37) |  |  | SRS (11), WBRT (5), focal RT (49), RT and SRS (2) | Not specified | Not specified |
|  |  | |  |  |  |  |  |  |
|  |  | |  |  |  |  |  |  |
| Tewarie et al., World Neurosurgery, 2022 | Following resection - WBRT (18), SRS (26), WBRT and SRS (8); radiation only – WBRT (25), SRS (48), WBRT and SRS (41) | | CTx (36), CPI (10) or TT (18) |  |  | SRS (26), SRS and WBRT (8), WBRT (18) | Not specified | Not specified |
|  |  | |  |  |  |  |  |  |
| Wasilewski et al., 2023 | Any RT modality (12) | | CTx and RTx (15), RT and TT (9), RT and CPI (17) |  |  | RT (6), SRS (40), WBRT (14) | Not specified | Not specified |
