# Supplementary material for: Re-resection of brain metastases – outcomes of an institutional cohort study and literature review
Source: BMC Cancer. 2025 Jun 1;25:973. doi: 10.1186/s12885-025-13677-0 (PMC12128291; doi:10.1186/s12885-025-13677-0)
Supplement: Supplementary file 5 — Additional file 5. Supplementary Table 3. Literature review of studies on secondary resection of recurrent brain metastases. Literature review of published studies (n=6 including this study) in the field of recurrent brain metastases that were treated with secondary resection, including details of various treatment characteristics. All studies were single-center studies. [file 12885_2025_13677_MOESM5_ESM.docx]

| **Publication** | **Median icPFS in months (IQR)** | | **Median time-to-recurrence resection in months (IQR)** | **Median ecPFS in months (IQR)** |  |  | **Median OS (IQR)** | **Median survival time after re-resection (IQR)** |
| --- | --- | --- | --- | --- | --- | --- | --- | --- |
| Bindal et al., Journal of Neurosurgery, 1995 | 6.7 (1.2-28.8) | | Not specified | Not specified |  |  | Not specified | 11.5 (7.8-14.0) |
| Schackert et al., Acta Neurochirurgica, 2013 | 6 (1-82.8) | | Not specified | Not specified |  |  | 7.5 (6.2-8.8) | Not specified |
| Kennion and Holliman, British Journal of Neurosurgery, 2017 | 7 (0-26.5 range) | | Not specified | Not specified |  |  | 18 (4.2-50.9 range) after OP1 | 7.6 (0.2-31.3 range) |
|  |  |  |  |  |  |  |  |  |
| Heßler et al., BMC Cancer, 2022 | 7.1 (5.8-8.2) | | Not specified | Not specified |  |  | 11.1 (8.4-13.6) after OP1 | Not specified |
|  |  | |  |  |  |  |  |  |
|  |  | |  |  |  |  |  |  |
| Tewarie et al., World Neurosurgery, 2022 | Median time to 2^nd^ recurrence = 7 | | Not specified | Not specified |  |  | Not specified | Not specified |
|  |  | |  |  |  |  |  |  |
| Wasilewski et al., 2023 | 7.7 (6.5-11.2) | | 11.6 (9.07-15.3) | 29.1 (16.5-NA) (data now shown in this manuscript) |  |  | 30.8 (20.4-51.6); 29.3 for patients with local recurrence and 34.7 for patients with distant recurrences | 11 (5.6-20.4) |
